# Ventricular Distribution Pattern of the Novel Sympathetic Nerve PET Radiotracer ^18^F-LMI1195 in Rabbit Hearts

**DOI:** 10.1038/s41598-019-53596-2

**Published:** 2019-11-19

**Authors:** Rudolf A. Werner, Hiroshi Wakabayashi, Xinyu Chen, Nobuyuki Hayakawa, Constantin Lapa, Steven P. Rowe, Mehrbod S. Javadi, Simon Robinson, Takahiro Higuchi

**Affiliations:** 1 The Russell H. Morgan Department of Radiology and Radiological Science, Division of Nuclear Medicine and Molecular Imaging, Johns Hopkins School University of Medicine, Baltimore, MD United States; 2 Department of Nuclear Medicine, University Hospital, University of Würzburg, Würzburg, Germany; 3 Department of Nuclear Medicine, Hannover Medical School, Hannover, Germany; 4 Comprehensive Heart Failure Center, University Hospital, University of Würzburg, Würzburg, Germany; 5 Department of Nuclear Medicine, University Hospital Augsburg, Augsburg, Germany; 6 Lantheus Medical Imaging, North Billerica, MA United States; 7 Department of Biomedical Imaging, National Cardiovascular and Cerebral Research Center, Suita, Japan; 8 Okayama University Graduate School of Medicine, Dentistry and Pharmaceutical Sciences, Okayama, Japan

**Keywords:** Cardiovascular diseases, Heart failure

## Abstract

We aimed to determine a detailed regional ventricular distribution pattern of the novel cardiac nerve PET radiotracer ^18^F-LMI1195 in healthy rabbits. *Ex-vivo* high resolution autoradiographic imaging was conducted to identify accurate ventricular distribution of ^18^F-LMI1195. In healthy rabbits, ^18^F-LMI1195 was administered followed by the reference perfusion marker ^201^Tl for a dual-radiotracer analysis. After 20 min of ^18^F-LMI1195 distribution time, the rabbits were euthanized, the hearts were extracted, frozen, and cut into 20-μm short axis slices. Subsequently, the short axis sections were exposed to a phosphor imaging plate to determine ^18^F-LMI1195 distribution (exposure for 3 h). After complete ^18^F decay, sections were re-exposed to determine ^201^Tl distribution (exposure for 7 days). For quantitative analysis, segmental regions of Interest (ROIs) were divided into four left ventricular (LV) and a right ventricular (RV) segment on mid-ventricular short axis sections. Subendocardial, mid-portion, and subepicardial ROIs were placed on the LV lateral wall. ^18^F-LMI1195 distribution was almost homogeneous throughout the LV wall without any significant differences in all four LV ROIs (anterior, posterior, septal and lateral wall, 99 ± 2, 94 ± 5, 94 ± 4 and 97 ± 3%LV, respectively, n.s.). Subepicardial ^201^Tl uptake was significantly lower compared to the subendocardial portion (subendocardial, mid-portion, and subepicardial activity: 90 ± 3, 96 ± 2 and *80 ± 5%LV, respectively, *p < 0.01 vs. mid-portion). This was in contradistinction to the transmural wall profile of ^18^F-LMI1195 (90 ± 4, 96 ± 5 and 84 ± 4%LV, n.s.). A slight but significant discrepant transmural radiotracer distribution pattern of ^201^Tl in comparison to ^18^F-LMI1195 may be a reflection of physiological sympathetic innervation and perfusion in rabbit hearts.

## Introduction

On a subcellular level, alterations of the myocardial sympathetic nervous system (SNS) in Heart Failure (HF) patients are predominantly driven by cardiac norepinephrine (NE) spillover^1^. This phenomenon provokes severe damage to cardiac myocytes, mainly by AMP-mediated calcium overload, and is caused by either elevated plasma concentration of NE, an impaired function of the NE transporter (NET, uptake-1), or reduced plasma clearance of NE in the synaptic cleft^1,2^. Recent years have witnessed the more expanded use of cardiac SNS-based imaging utilizing such NET ligands for either single photon emission computed tomography (SPECT) or positron emission tomography (PET) technology, which both allow for a thorough evaluation of sympathetic neurotransmission in HF patients^3–9^.

Comparable to their physiological counterpart, those radiolabeled analogs of NE share similar pathways and are also taken up by the uptake-1 mechanism, which clears exocitotically released NE from the synaptic cleft^3^. In the last decade, several cardiac PET radiotracers have been demonstrated to precisely reflect sympathetic nerve integrity in a clinical setting: The prospective Prediction of ARrhythmic Events with Positron Emission Tomography (PAREPET) trial showed that the neuronal SNS PET agent ^11^C-Hydroxyephedrine (^11^C-HED) identifies HF patients who are at highest risk for sudden cardiac arrest or death^10^. While ^11^C-HED has been extensively used to investigate cardiac sympathetic nerve conditions, its high cost and the necessity of staff to prepare such short-half-life radiopharmaceuticals limit its widespread adoption^3^. This is in contradistinction to ^18^F-labeled SNS radiotracers, such as ^18^F-LMI1195, which do not require costly on-site preparation due to the longer half-life of ^18^F (110 min). Thus, dispatch from central cyclotron facilities, even by commercial vendors, is feasible and such an approach has been proven to be profitable for the most commonly used oncology imaging agent, 2-deoxy-2-^18^F-fluoro-d-glucose^3,11^.

Moreover, in a recently published phase-2 study, ^18^F-LMI1195 demonstrated superior kinetics compared to ^11^C-HED, in particular for early imaging time-points. Thus, a phase-3 trial in ischemic cardiomyopathy patients scheduled to receive an implantable cardioverter defibrillator (ICD) is underway and may further corroborate the potential benefit of ^18^F-LMI1195 for risk stratification^12^. However, if this PET agent will one day be routinely available in the clinic to help identify high-risk patients and support clinical decision-making, a more precise understanding of the subcellular radiotracer handling may be fundamentally necessary for the interpretation of imaging results. Notably, previous studies have already proven the high affinity of ^18^F-LMI1195 towards neuronal uptake-1 along with a stable storage in presynaptic vesicles^13,14^. However, further insights into the normal regional ventricular distribution of such radiotracers are essential for designing clinical protocols and comprehension of imaging findings. Thus, in the present study, we aimed to elucidate the distribution pattern of ^18^F-LMI1195 over the left ventricular (LV) wall in healthy rabbits.

## Material and Methods

Animal protocols were approved by the local Animal Care and Use Committee (National Cardiovascular and Cerebral Research Center, Suita, Japan) and conducted according to the Guide for the Care and Use of Laboratory Animals (NIH Publication No. 85-23, revised 1996)^15^.

### Radiopharmaceuticals

^18^F-LMI1195 was synthesized following described procedures^16,17^. Analyses at the end of syntheses revealed specific radioactivity greater than 100 GBq/μmol and radiochemical purity greater than 95% for the radiolabeled compound.

### Dual-radiotracer autoradiography

**Figure 1:**
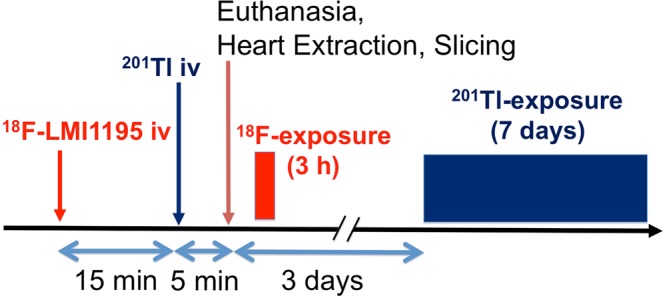
Schematic diagram illustrating the protocol for the dual-radiotracer autoradiography analysis using ^18^F-LMI1195 and ^201^Tl.

In healthy New Zealand White rabbits weighing 3.8–4.2 kg (n = 4), *ex-vivo* high resolution autoradiographic imaging was conducted to determine accurate ventricular distribution of ^18^F-LMI1195. As a reference, the myocardial perfusion marker ^201^Tl was co-injected for dual-tracer analysis. ^18^F-LMI1195 (25 MBq) was injected via ear vein followed by ^201^Tl (0.5 MBq). Rabbits were euthanized 20 min after ^18^F-LMI1195 administration, and the heart was removed. The heart was sliced into 20 μm thickness slices for autoradiographic analysis. The short axis sections were exposed to a phosphor imaging plate (MultiSensitive phosphor screen; PerkinElmer, Shelton, CT) to determine ^18^F-LMI1195 distribution (exposure for 3 h) with a digital autoradiography system (CR 35 Bio, Raytest or Cyclone; Packard; Straubenhardt, Germany). After complete ^18^F decay of 3 days, sections were again re-exposed to determine ^201^Tl distribution (second exposure for 7 d). Figure 1 displays the described experimental setup.

### Quantitative analysis

In order to quantify radiotracer uptake distribution, regions of interest (ROIs) were drawn on four left ventricular (LV) segments and a right ventricular (RV) segment on a mid-ventricular short axis section. To assess the transmural pattern of radiotracer distribution in the ventricular wall, the ROIs were divided into subepicardial, mid-portion, and subendocardial wall portions on the slices^18^.

### Statistical analysis

Results are presented as means ± standard deviation (SD). The two-tailed paired Student’s t-test was used to compare differences between two dependent groups and the two-tailed independent Student’s t-test between independent groups. Values of p < 0.05 were considered statistically significant.

## Results

### Assessment of ^18^F-LMI1195 distribution throughout the LV Wall

^18^F-LMI1195 distribution was homogeneous throughout the LV wall and no significant differences in radiotracer activity were detected in all short axis LV ROIs (anterior, posterior, septal and lateral walls, 99 ± 2, 94 ± 5, 94 ± 4 and 97 ± 3%LV, n.s.).

### Comparison of ventricular distribution patterns for both radiotracers

**Figure 2:**
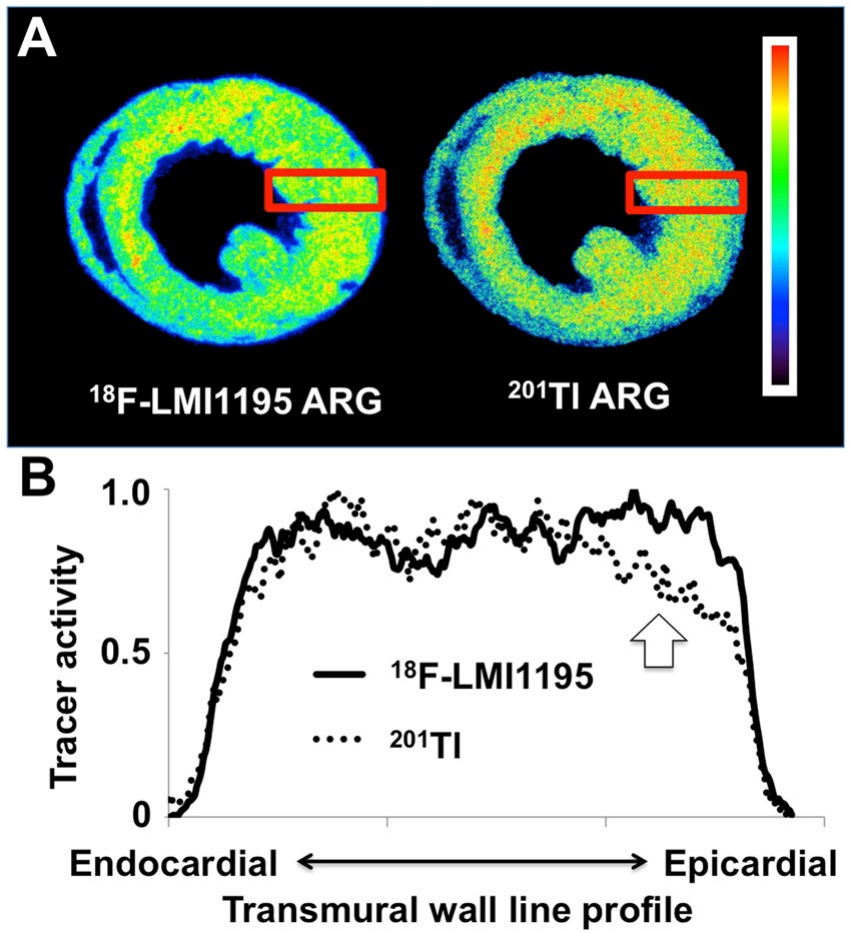
(**A**) Representative cross-sectional short-axis images at a midventricular level on dual-radiotracer autoradiography with ^18^F-LMI1195 and ^201^Tl. ^18^F-LMI1195 distribution was homogeneous throughout the left ventricular wall and no significant differences in radiotracer activity were detected in mid-ventricular short axis slices. This is in contradistinction to the autoradiography (ARG) of ^201^Tl: A slight discrepant uptake pattern in the subepicardial wall portion can be appreciated compared to ^18^F-LMI1195 ARG. (**B**) Transmural wall line profile of both ^18^F-LMI1195 and ^201^Tl. The macroscopic findings were further corroborated quantitatively: Compared to the subendocardial wall section, radiotracer activity for ^201^Tl was significantly lower in the subepicardial wall portion. ^18^F-LMI1195 remained stable over all three section (subendocardial portion, mid-portion, and subepicardial portion).

^201^Tl uptake in the subepicardial portion was significantly lower than in the subendocardial portion (subendocardial, mid-portion and subepicardial activity: 90 ± 3, 96 ± 2 and *80 ± 5%LV, respectively, *p < 0.01 vs. mid-portion). Those findings were in contradistinction to the cardiac SNS radiotracer: ^18^F-LMI1195 demonstrated no significant differences between the three portions (90 ± 4, 96 ± 5, and 84 ± 4%LV, respectively, n.s.). Representative cross-sectional short-axis images at a midventricular level on dual-radiotracer autoradiography with ^18^F-LMI1195 and ^201^Tl are displayed in Fig. 2A. The ventricular distribution patterns for ^18^F-LMI1195 and ^201^Tl are highlighted in a transmural wall line profile (from endo- to epicardial wall portions, Fig. 2B): a decline in radiotracer activity for the reference perfusion marker at the epicardial section can be appreciated, while ^18^F-LMI1195 remained stable over all three wall portions.

## Discussion

The present study investigated the transmural distribution pattern of the novel SNS imaging agent ^18^F-LMI1195 in comparison to myocardial perfusion assessed by ^201^Tl in healthy rabbits. Using *ex vivo* high resolution autoradiographic imaging, which is free from *in vivo* imaging artifacts (e.g., motion, attenuation, and partial volume effect), a slight but significant discrepant transmural radiotracer distribution pattern of the perfusion agent in comparison to ^18^F-LMI1195 was noted: ^201^Tl uptake in the subepicardial section was significantly lower than subendocardial, while the SNS agent demonstrated no significant differences over all three wall portions.

The prospective PAREPET trial demonstrated that denervated myocardium assessed by ^11^C-HED serves as a strong outcome predictor for sudden cardiac arrest or death in high-risk HF patients^10^. However, owing to their short half-life, such ^11^C-labeled radiotracers suffer from several drawbacks. These include, but are not limited to a potential cold-mass effect (decrease of NE re-uptake function at higher amount of cold mass), the need for a costly on-site cyclotron, and the necessity for expert staff to prepare such radiopharmaceuticals^3,4^. Thus, novel ^18^F-labeled radiotracers for mapping cardiac nerve integrity, such as ^18^F-LMI1195, may overcome these hurdles: their longer half-life may allow for radiotracer distribution from central cyclotron facilities and flexibility in study design, e.g., by facilitating delayed imaging protocols^7^.

An extensive body of evidence has reported on the high affinity of ^18^F-LMI1195 for neuronal uptake-1 including cell-membrane binding assays and *in vitro* blocking studies with the potent uptake-1 blocker desipramine^13,14^. In addition, a desipramine chase protocol (delivery after radiotracer injection) led to no increased radiotracer washout, which indicates stable storage in presynaptic nerve terminals. Those findings were further confirmed in NE-expressing, vesicle-poor and vesicle-rich cell lines as well as in an isolated perfused rabbit heart setup using electric field stimulation (as vesicle turnover stimulant)^13,19^. Altogether, ^18^F-LMI1195 is taken up via uptake-1 and stably stored inside presynaptic vesicles and thus, this catecholamine analogue closely mimicks physiological NE turnover. Adding to those previous described findings, the present study provides further insight into the ventricular distribution of ^18^F-LMI1195 over the LV wall: *ex vivo* high resolution autoradiographic imaging demonstrated not only homogeneous radiotracer distribution in the LV, but also no significant differences in subendocardial, mid-portion, and subepicardial activity. Thus, the transmural wall line profile of ^18^F-LMI1195 further corroborates a precise reflection of cardiac sympathetic neurotransmission with this radiotracer. Notably, all of those previously mentioned studies investigating ^18^F-LMI1195 in a preclinical setting have been conducted using rabbit myocardium: similar to the human heart, NE clearance through neuronal uptake-1 is also pronounced in the rabbit heart. While extrapolations from animals to humans have to be made with caution, the rabbit myocardium may provide a reliable platform to investigate ^18^F-LMI1195 radiotracer distribution, as it closely mimicks human cardiac nerve integrity^20^. The findings in this study may, therefore, have direct applicability to the clinical setting.

In a transient myocardial ischemia model using Wistar rats, a higher susceptibility of cardiac sympathetic neurons compared to cardiomyocytes was demonstrated: in a head-to-head comparison of ^201^TI and ^11^C-HED, a larger defect for the latter radiotracer was noted and such uptake defect areas corresponded to the histologically identified regions of denervation. In addition, partial re-innervation in the chronic phase was demonstrated (shown by recovery of subepicardial ^11^C-HED uptake), while no such findings were observed for ^201^TI^18^. Further adding to the complexity of this phenomenon, post-infarction sympathetic neuronal damage in humans assessed by ^123^I-metaiodobenzylguanidine (^123^I-mIBG) also exceeded the infarct size (assessed by ^99m^Tc-sestamibi SPECT)^21^. In a prospective setting, *Nishisato et al*. enrolled 160 patients with consecutive ICDs and compared the myocardial SNS conditions using ^123^I-mIBG to myocardial perfusion alterations with ^99m^Tc-tetrofosmin. This constituted a combined assessment of cardiac nerve and perfusion status and was of superior benefit in predicting ICD discharge rate than one imaging test alone^22^. Estorch *et al*. reported that in patients who developed angina during exercise test, the area of denervated but perfused myocardium was significantly larger than in subjects without angina^23^. Matsunari and coworkers reported similar findings: Sympathetic neuronal damage measured by ^123^I-mIBG was larger than the infarct size assessed by perfusion imaging, suggesting higher susceptibility of sympathetic neurons compared to cardiac myocytes^21^. Integrating those available data, the precise assessment of the transmural wall line profile of different cardiac nerve radiotracers compared to perfusion references in both healthy and impaired myocardium are of value. Thus, if a more widespread adoption of such dual-radiotracer approaches is to be pursued in a clinical setting in the near future, the properties of ^18^F-LMI1195 described in this manuscript may be of importance for interpreting imaging results of perfusion status and cardiac neurotransmission in the failing heart. In the present study, no *in vivo* PET study has been carried out. However, previous investigations have already proven the feasibility of *in-vivo* ^18^F-LMI1195 PET imaging using the identical animal model of New Zeland Rabbits and in humans^14,17^. Apart from that, the herein obtained results may lay the groundwork to study both cardiac nerve integrity and myocardial perfusion in dedicated animal models of myocardial ischemia, preferably by using an ^18^F-labeled SNS imaging agent like LMI1195.

## Conclusions

In healthy rabbits, a significant discrepant transmural radiotracer distribution pattern of ^201^Tl in comparison to ^18^F-LMI1195 may be a reflection of physiological cardiac sympathetic innervation and perfusion. If ^18^F-LMI1195 will one day be routinely available in the clinic, the herein presented distinct characteristics in the LV distribution pattern of perfusion status and cardiac nerve integrity may lay the groundwork for a more thorough evaluation of imaging results.
